# Genomic Assays for Identification of Chikungunya Virus in Blood Donors, Puerto Rico, 2014

**DOI:** 10.3201/eid2108.150458

**Published:** 2015-08

**Authors:** Charles Y. Chiu, Vanessa Bres, Guixia Yu, David Krysztof, Samia N. Naccache, Deanna Lee, Jacob Pfeil, Jeffrey M. Linnen, Susan L. Stramer

**Affiliations:** University of California San Francisco, San Francisco, California, USA (C.Y. Chiu, G. Yu, S.N. Naccache, D. Lee, J. Pfeil);; University of California San Francisco–Abbott Viral Diagnostics and Discovery Center, San Francisco (C.Y. Chiu, G. Yu, S.N. Naccache, D. Lee, J. Pfeil);; Hologic, Inc., San Diego, California, USA (V. Bres, J.M. Linnen);; American Red Cross, Gaithersburg, Maryland, USA (D. Krysztof, S.L. Stramer)

**Keywords:** chikungunya virus, transfusion-transmitted infection, blood donor, ViroChip microarray, next-generation sequencing, whole-genome sequencing, transcription-mediated amplification, viruses

## Abstract

A newly developed transcription-mediated amplification assay was used to detect chikungunya virus infection in 3 of 557 asymptomatic donors (0.54%) from Puerto Rico during the 2014–2015 Caribbean epidemic. Viral detection was confirmed by using PCR, microarray, and next-generation sequencing. Molecular clock analysis dated the emergence of the Puerto Rico strains to early 2013.

Chikungunya virus (CHIKV), a mosquitoborne alphavirus, family *Togaviridae*, causes an acute illness, manifested as fever and severe arthralgia (*1*). CHIKV infections are associated with global epidemics, and cases reemerged in the Americas in December 2013 after an ≈200-year absence (*1*,*2*). The initial cases were reported from the island of Saint Martin in the Caribbean, with autochthonous cases reported across 9 islands by April 2014. By March 2015, >1,250,000 suspected or confirmed cases had been reported in the Americas (http://www.paho.org/hq/index.php?Itemid=40931), including ≈3,200 cases in North America, most (89%) in returning travelers.

Risk for transfusion-transmitted infection (TTI) of CHIKV is currently unclear. However, several factors raise concern about possible CHIKV TTI, including a 10%–25% asymptomatic infection rate and high viremic titers in asymptomatic persons (*3*). Recently, a probable TTI case from Ross River virus (RRV), an alphavirus related to CHIKV, was reported in a person who had received RRV-positive donor blood, and a clinically compatible illness developed with subsequent seroconversion (*4*).

## The Study

**Figure 1:**
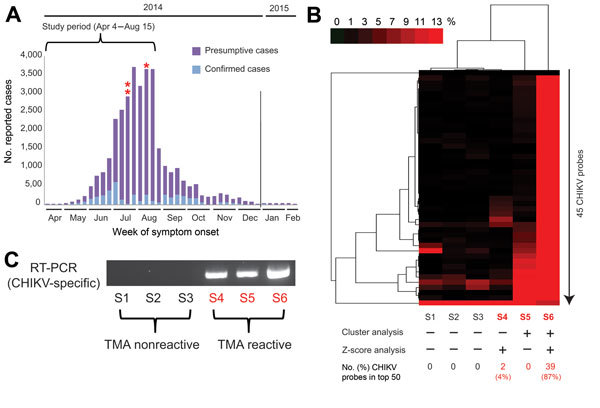
New genomic tests for chikungunya (CHIKV) infection in blood donors. A) Epidemic curve of reported cases in Puerto Rico, April 2014–February 2015. For 2014, 30,983 presumptive cases and 4,275 laboratory-confirmed cases were reported to the Secretary of Health in Puerto Rico. Three CHIKV-positive case-patients (asterisks) of 557 tested were identified by transcription-mediated amplification (TMA) screening of plasma samples during the study period. B) Heat map (cluster analysis) of 6 ViroChip (University of California San Francisco, San Francisco, CA, USA) microarrays corresponding to 6 donor plasma samples, 3 CHIKV positive and 3 CHIKV negative. Only microarray probes derived from CHIKV are plotted because signatures for other bloodborne viral pathogens were absent (data not shown). A sample is called ViroChip positive for CHIKV if at least 10% of the CHIKV probes on the heat map have a normalized probe intensity of >10% by cluster analysis (*5*) and/or if >1 probe is detected within the top 50 by *z* score analysis (*6*). Red bar denotes the magnitude of hybridization intensity normalized across the 45 CHIKV probes on the microarray. ViroChip microarray data have been submitted to the Gene Expression Omnibus database repository (accession no. GSE67234). C) Reverse transcription PCR (RT-PCR) testing for CHIKV and visualization of the PCR amplicon by 2% agarose gel electrophoresis confirm the transcription-mediated and ViroChip microarray results (*7*). D) Metagenomic next-generation sequencing (NGS) of the 3 CHIKV-positive plasma samples enables recovery of the viral genome. For each sample, coverage plots of mapped NGS reads to the “best hit” viral genome (accession no. KJ451624), identified by using the automated sequence-based ultrarapid pathogen identification pipeline, are shown (*8*). The read coverage (*y* axis, log scale) is plotted as a function of nucleotide position along the genome (*x* axis). The consensus whole-genome sequences obtained from the coverage plots are used for the subsequent phylogenetic and molecular clock analyses (Figure 2). NGS reads with human sequences removed have been deposited in the Sequence Read Archive (BioProject accession no. PRJNA282046; SRP accession no. SRP057614). The 3 CHIKV genome sequences have been deposited in GenBank (accession nos. KR264949–KR264951).

A prototype CHIKV transcription-mediated amplification (TMA) assay was used to screen blood donors from Puerto Rico during the peak of the 2014 Caribbean epidemic (Table). After routine blood donation to the American Red Cross April 4–August 14, 2014, frozen surplus plasma samples from all donors were de-identified and retained for study (all collected during the peak weeks of the 2014 CHIKV outbreak; http://www.salud.gov.pr/Estadisticas-Registros-y-Publicaciones/Pages/Chikungunya.aspx) (Figure 1, panel A). Each retained sample tested negative for pathogens on all required donation screening tests and was also negative for investigational dengue virus (DENV, types 1–4) RNA by TMA (*8*). Passive reporting was encouraged by use of a donor information sheet describing signs/symptoms of DENV and CHIKV infection. No donor reported any symptoms of arbovirus infection from the time of collection through 12 days following donation. The 557 samples were screened with a candidate screening real-time TMA CHIKV assay with a 95% limit of detection of 16.27 RNA copies/mL (95% CI 11.10–29.56 copies/mL) on the high-throughput automated Panther system (Hologic, Inc., San Diego, CA, USA). Each sample was tested in singlet; reactive samples were diluted 1:16 and logarithmically from 10^−2^ to 10^−8^ and retested in triplicate. Three samples (0.54%) were CHIKV RNA–reactive by TMA, with estimated viral loads ranging from 2.9 × 10^5^ to 9.1 × 10^7^ copies/mL (Table). One sample corresponded to a donor who had a confirmed diagnosis of CHIKV infection when contacted after the 12-day reporting period (7.6 × 10^5^ copies/mL); the other 2 donors remained asymptomatic.

**Table:** Asymptomatic blood donors testing positive for CHIKV infection, Puerto Rico, 2014*

Collection date, 2014	Prototype CHIKV real-time assay on Panther system, dilution†‡								
	Initial testing, undiluted	Confirmatory testing							
		1:16	1:100	1:1,000	1:10^4^	1:10^5^	1:10^6^	1:10^7^	1:10^8^
Jul 15									
Reactive/total no. tested	1/1	3/3	3/3	3/3	3/3	2/3	0/3	NT	NT
Estimated copies/mL	2.9 × 10^5^								
Jul 16§									
Reactive/total no. tested	1/1	3/3	3/3	3/3	3/3	3/3	2/3	NT	NT
Estimated copies/mL	7.6 × 10^5^								
Aug 14									
Reactive/total no. tested	1/1	3/3	3/3	2/2	3/3	3/3	3/3	3/3	3/3
Estimated copies/mL	9.1 × 10^7^								

*CHIKV, chikungunya virus; NT, not tested. †For the real-time CHIKV, transcription-mediated amplification assay, plasma samples (0.5 mL) were tested on the fully automated Panther system which performs magnetic target specific capture, amplification, and real-time detection in the presence of an internal control. During the target capture step, the hybridized target is captured onto magnetic micro-particles that are separated from the specimen in a magnetic field. Wash steps remove extraneous components from the reaction tube. Target amplification occurs by using 2 enzymes, MMLV (Moloney murine leukemia virus) reverse transcription and T7 RNA polymerase. Detection is achieved using single-stranded fluorescent labeled nucleic acid probes that are present during the amplification of the target. The time for the fluorescent signal to reach a specified threshold is proportional to the starting CHIKV RNA concentration. The primers, detection probes, and target capture oligonucleotides hybridize to highly conserved regions of CHIKV RNA genome and were designed to detect all 3 major CHIKV lineages. The cutoff for reactive reactions was set by the investigators at 1,000 relative fluorescent units. Estimated copies per mL were calculated relative to the emergence time of the emitted fluorescence of a calibration curve generated by logarithmic dilution of a CHIKV in vitro synthetized transcript. ‡Dilutions were performed in defribrinated, pooled plasma, passed through a 0.2-μm filter, dialyzed to approximate a human serum profile, delipidated for clarity/stability, and prescreened as nonreactive for CHIKV. §CHIKV-positive donor reported postdonation fever and joint pain at 2 d postdonation.

For confirmation, we performed blinded orthogonal panviral microarray (ViroChip, University of California San Francisco, San Francisco, CA, USA) and PCR testing of 6 samples, the 3 positive for CHIKV and 3 randomly selected negative controls. (ViroChip is a DNA-detection microarray containing 57,444 probes, and the latest version (v. 5.0) represents all viruses in GenBank as of December 2010 [*9*]). Nucleic acid extraction was performed from 400 μL of TRIzol-inactivated donor serum by using the Direct-zol RNA MiniPrep Kit (Zymo Research, Irvine, CA, USA), and on-column treatment was performed with Turbo DNase (Life Technologies, Carlsbad, CA, USA). After microarray processing, ViroChip hybridization patterns were analyzed by using hierarchical clustering and z-score analysis (*6*). Each of the 3 TMA-positive samples was positive for CHIKV by ViroChip by one or both analysis methods (Figure 1, panel B), whereas all 3 controls tested negative by ViroChip. Given the presence of sparse cross-hybridization artifacts in individual microarray probes (Figure 1, panel B), we further tested the samples using a previously reported CHIKV PCR assay (*7*), which generated results 100% concordant with those of TMA (Figure 1, panel C).

**Figure 2:**
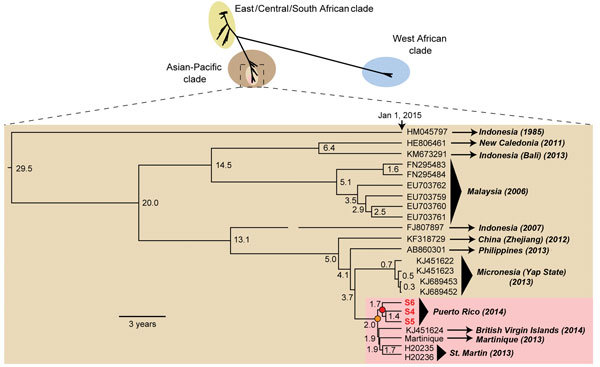
Phylogeny of chikungunya virus (CHIKV). (Upper panel) All 188 nearly-full or full genome CHIKV sequences available in the National Center for Biotechnology Information nucleotide database as of March 2015, including the 3 new genomes from Puerto Rico recovered in this study (red boldface) were aligned by using the multiple alignment fast Fourier transform (MAFFT) algorithm, and phylogenetic trees were constructed by using the MrBayes algorithm in the Geneious software package (*12*). Branch lengths are drawn proportionally to the number of nucleotide substitutions per position, and support values are shown for each node. (Lower panel) Molecular clock analysis of the Southeast Asian/Pacific branch containing the Caribbean sublineage (pink) was performed by using BEAST software (*13*). Branch lengths are drawn proportionally to the number of years before January 1, 2015, and the number of years is shown for each node. The 3 major lineages and Caribbean-associated sublineage are shown in different colors, and the nodes corresponding to the Caribbean (orange) and Puerto Rico (red) offshoots are highlighted.

We then used unbiased metagenomic next-generation sequencing (NGS) (*9*) as a pan-pathogen screen and to recover the viral genome from the 3 CHIKV-positive samples (Figure 1, panel D). NGS libraries were constructed by using the Nextera XT kit (Illumina, San Diego, CA, USA) and validated as described (*10*), followed by 161-bp, single-end sequencing on an Illumina MiSeq instrument. Raw NGS data (3.2–32.4 million reads per sample) were analyzed for reads corresponding to known pathogens by using the sequence-based ultrarapid pathogen identification (SURPI) computational pipeline (*10*). After computational subtraction of human host reads, alignment was performed against all microbial sequences in the National Center for Biotechnology Information nucleotide database and the best hit selected on the basis of percentage of mapped read coverage and pairwise identity. A Caribbean strain of CHIKV from the British Virgin Islands (accession no. KJ451624) (*11*) was identified by SURPI as the closest matching viral genome; 95%–100% genome coverage was obtained for the 3 CHIKV-positive donors (Figure 1, panel D). Phylogenetic analysis of the 3 Puerto Rico CHIKV genomes, together with all 188 publicly available sequenced CHIKV genomes in the reference database, placed the Puerto Rico strains in the Caribbean clade (Figure 2, panels A, B). Molecular clock analysis revealed that this clade, an offshoot of the Southeast Asian/Pacific lineage, possibly recently emerged in the Western Hemisphere in early 2013, with the 3 Puerto Rico viruses diverging from the other Caribbean strains 1.7 years ago (Figure 2, panel C).

## Conclusions

We employed several orthogonal genomic-based assays to detect CHIKV infection by real-time TMA testing in 3 asymptomatic donors during the peak of the 2014–2015 Caribbean epidemic (*1**,**2*). We confirmed this finding using specific PCR, microarray, and NGS analyses (*9*) and tracked the emergence of CHIKV in the Western Hemisphere to early 2013 by NGS-based whole-genome sequencing and molecular clock analysis. The rate of CHIKV positivity in donors from Puerto Rico (3/557, 0.54%) is slightly higher than that previously reported in donors from the French West Indies (4/2,149, 0.2%; p = 0.16 by Fisher 2-tailed exact test) (*14*). In that study, 2 of 4 CHIKV-positive donors had febrile illness 12–24 hours postdonation, whereas fever and joint pains developed in 1 of 3 CHIKV-positive donors in our study. The level of viral RNA in the Puerto Rico donor from with the highest titer, 9.1 × 10^7^ copies/mL, who remained asymptomatic, is comparable to the median viral titer observed previously in symptomatic CHIKV patients (5.6 × 10^5^ PFU/mL, or ≈5.6 × 10^7^ copies/mL) (*3*). No cases of CHIKV-associated TTI have been confirmed to date, although potential transmission by that route of related alphaviruses such as RRV has been documented (*4*). Nevertheless, our results indicate that high-titer asymptomatic CHIKV infections, if transmissible by transfusion, can readily elude routine screening based solely on postdonation reporting of febrile illness.

New genomic-based technologies have utility for outbreak investigation, bloodborne pathogen screening, and disease surveillance (*9*). The availability of a high-throughput TMA assay will facilitate screening for CHIKV and more precisely establish the risk of transfusion-associated transmission. Panviral microarrays are useful for broad surveillance of bloodborne pathogens (*9*), yet rigorous individual probe validation across multiple targets is needed because of potential cross-hybridization artifacts. Metagenomic NGS (*9**,**10*) is an unbiased diagnostic method that identifies all potential pathogens simultaneously on the basis of uniquely identifying DNA sequences. In our study, metagenomic NGS and SURPI analysis facilitated rapid identification and whole-genome recovery of 3 Puerto Rican CHIKV strains directly from primary samples without the need for viral culture. 

Recovery from CHIKV infection appears to confer lifelong immunity, and thus an unknown but potentially large fraction of the population of the Puerto Rico may be immune. However, the ongoing threat to returning travelers and spread of the mosquito vector to immunologically naive populations (e.g., in United States and Mexico) underscore the need for continual donor surveillance (*15*). Increased use of microarrays and NGS in the future would be anticipated, given its suitability for detecting threats from multiple emerging vector-borne diseases such as chikungunya and dengue (*2*).
